# Comparison of acute kidney injury following brain death between male and female rats

**DOI:** 10.1016/j.clinsp.2023.100222

**Published:** 2023-05-31

**Authors:** Roberto Armstrong-Jr, Fernanda Yamamoto Ricardo-da-Silva, Marina Vidal-dos-Santos, Lucas Ferreira da Anunciação, Petra J. Ottens, Cristiano Jesus Correia, Luiz Felipe Pinho Moreira, Hendrik Gerrit Derk Leuvenink, Ana Cristina Breithaupt-Faloppa

**Affiliations:** a Department of Surgery, University Medical Centre Groningen, University of Groningen, Groningen, the Netherlands; b Laboratório de Cirurgia Cardiovascular e Fisiopatologia da Circulação (LIM-11), Instituto do Coração (InCor), Faculdade de Medicina da Universidade de São Paulo, São Paulo, SP, Brasil

**Keywords:** Brain death, Sex differences, Acute kidney injury, Isolated perfused kidney, Rat

## Abstract

**Background:**

Clinical reports associate kidneys from female donors with worse prognostic in male recipients. Brain Death (BD) produces immunological and hemodynamic disorders that affect organ viability. Following BD, female rats are associated with increased renal inflammation interrelated with female sex hormone reduction. Here, the aim was to investigate the effects of sex on BD-induced Acute Kidney Injury (AKI) using an Isolated Perfused rat Kidney (IPK) model.

**Methods:**

Wistar rats, females, and males (8 weeks old), were maintained for 4h after BD. A left nephrectomy was performed and the kidney was preserved in a cold saline solution (30 min). IPK was performed under normothermic temperature (37°C) for 90 min using WME as perfusion solution. AKI was assessed by morphological analyses, staining of complement system components and inflammatory cell markers, perfusion flow, and creatinine clearance.

**Results:**

BD-male kidneys had decreased perfusion flow on IPK, a phenomenon that was not observed in the kidneys of BD-females (*p* < 0.0001). BD-male kidneys presented greater proximal (*p* = 0.0311) and distal tubule (*p* = 0.0029) necrosis. However, BD-female kidneys presented higher expression of eNOS (*p* = 0.0060) and greater upregulation of inflammatory mediators, iNOS (*p* = 0.0051), and Caspase-3 (*p* = 0.0099). In addition, both sexes had increased complement system formation (C5b-9) (*p*=0.0005), glomerular edema (*p* = 0.0003), and nNOS (*p* = 0.0051).

**Conclusion:**

The present data revealed an important sex difference in renal perfusion in the IPK model, evidenced by a pronounced reduction in perfusate flow and low eNOS expression in the BD-male group. Nonetheless, the upregulation of genes related to the proinflammatory cascade suggests a progressive inflammatory process in BD-female kidneys.

## Highlights

• Experimental evidence suggested that hypoperfusion could impact organ viability.
• High eNOS-expression in female rats favored the maintenance of renal perfusion.
• BD-female rats showed progressive inflammatory responses.

## Introduction

Chronic Kidney Disease (CKD) is a common problem that may lead to irreversible renal failure. For patients with CKD, kidney transplantation provides improved life expectancy compared to dialysis. Unfortunately, while the incidence of end-stage renal disease patients increases, the quantity of viable donor kidneys is not sufficient to supply a long waiting list [1]. The majority of organs come from donors who have been diagnosed with brain death. Rebolledo et al. [2] reported that Brain Death (BD) in male rats led to renal deterioration, which was associated with low renal function, renal inflammation, and increased oxidative stress markers. Ferreira et al. [3] showed an important influence of sex on the consequences of BD, with higher proinflammatory status presented by female rats due to acute estradiol reduction after hypothalamic/pituitary failure. Additionally, estradiol absence is associated with poor outcomes in kidney transplantation in female recipient rats [4]. Clinical and experimental studies have focused on developing strategies to expand the supply of organs, including donor management, quality, and preservation of organs for transplantation [5,6].

Since BD induces hemodynamic, neurohumoral, and inflammatory changes [7], and sex hormones exert important modulatory effects on the inflammatory response [8], the authors aimed to evaluate sex differences in renal alterations generated by BD and kidney viability using in normothermic isolated perfused rat kidney model.

## Materials and methods

### Animals

Female and male Wistar F344/IcoCrl rats (8 weeks old) (Charles River, Italy), were kept in a 12h light-dark cycle at 23 ± 2°C and received water and food *ad-libitum*. The experimental protocol was approved (IvD 171245-01-002) by the local animal ethics committee according to the Experiments on Animals Act.

**Fig. 1:**
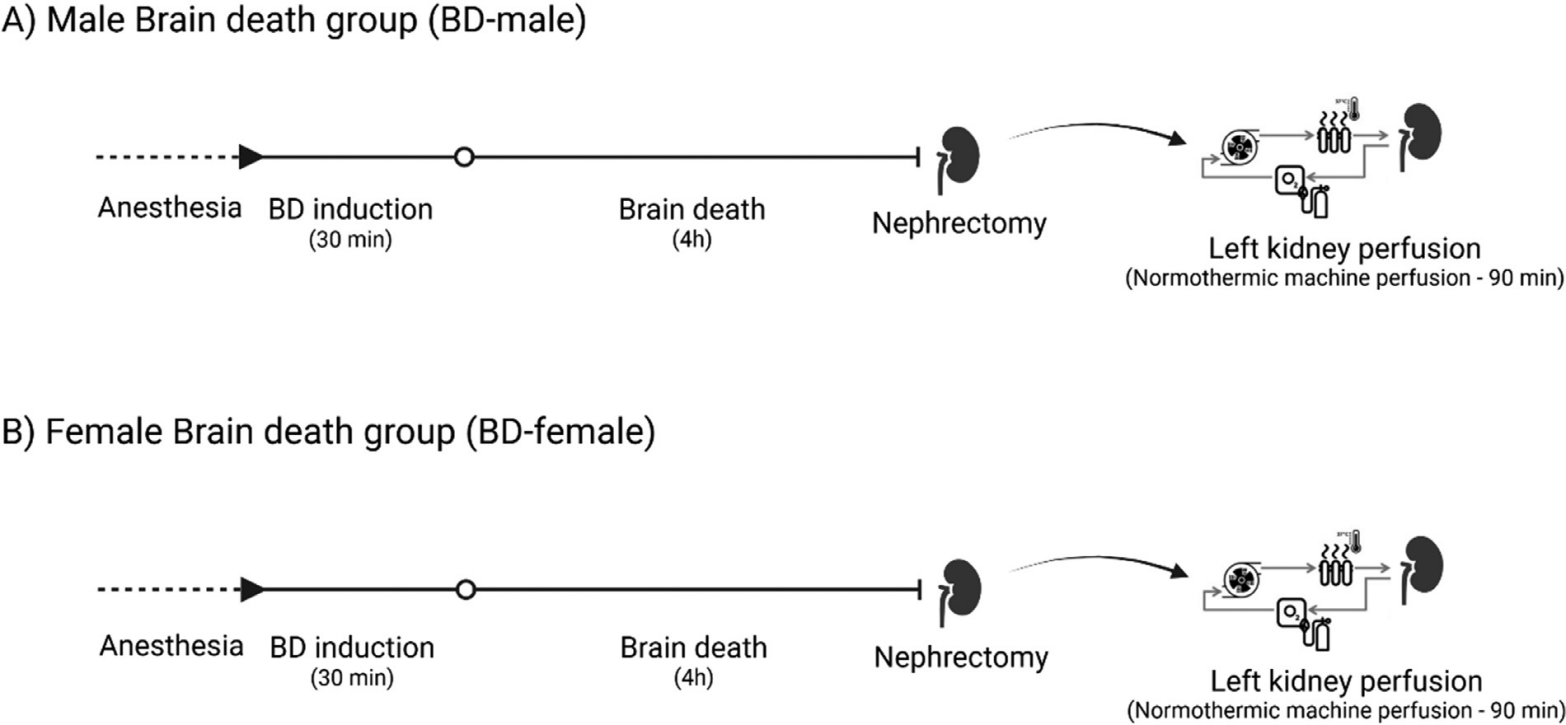
Experimental timeline. Male and Female rats were submitted to BD. At the end of 4h of BD experiment, left nephrectomy (LK) was performed for Isolated Kidney Perfusion (IPK – 90 min) and right nephrectomy for organ collection (RK).

Here, 32 rats were randomized and assigned to four groups (Fig. 1). For this study, female rats in the proestrus/estrus phases of the estrus cycle were used.

### Estrus cycle identification

Vaginal lavage was carried out with a Pasteur pipette filled with 10 µL of saline (NaCl 0.9%) and the obtained fluid was stained with crystal violet solution. The estrus/proestrus cycle was identified using an optical microscope.

### Brain death (BD) model

All rats were anesthetized with 2%–5% isoflurane and ventilated by tracheotomy using a rodent ventilator (frequency: 70 breaths/min, tidal volume: 10 mL/kg) (Harvard Apparatus, model 683, USA). The temperature was maintained at 37°C using a heating pad. Mean Arterial Pressure (MAP) was continuously regulated. The femoral vein was used to administer fluids for hemodynamic stabilization, and the cannulation of the carotid artery was performed for blood sampling and continuous arterial pressure examination.

BD model was based on Kolkert et al. [9]. Summarily, a skull hole was drilled to insert the 4F Fogarty balloon catheter (Edwards Lifesciences LLC, USA) into the epidural area through the anterolateral space and inflated slowly. BD induction was concluded after 30 min and confirmed by the apnea test. Following BD induction, anesthesia was stopped, and fluid administration was initiated, remaining for the duration of the experiment (4h).

At the end of the experiment, blood, urine, and the Right Kidney (RK) were collected. The RK was snap-frozen in liquid nitrogen or embedded in paraffin, and urine and serum were placed at -80°C. Following warm ischemia (30 min), Left Nephrectomy (LK) was performed for Isolated Kidney Perfusion (IPK). Then, the ureter and renal arteries were cannulated.

### Normothermic isolated perfused kidney (IPK) setup

The rat IPK setup has been previously reported [10]. Briefly, pressure-controlled IPK was performed using a roller pump (Ismatec ISM404, Switzerland). The perfusion solution contained 100 mL of William's Medium E (Life Technologies, USA) supplemented with 30 mmoL/L HEPES (Sigma-Aldrich, USA), 7 mmoL/L creatinine (Sigma-Aldrich, Netherlands), and 50 g/L bovine serum albumin fraction V (Sigma-Aldrich). The perfusion solution was oxygenated with a carbogen mixture (95% O_2_/5% CO_2_ at a flow rate of 0.5 L/min. The perfusion fluid temperature was maintained at 37°C.

Ultrafiltrated from the ureter and perfusion solution from the circuit samples were collected after 90 min of renal perfusion. After 90 min, the LK was snap-frozen in liquid nitrogen or embedded in paraffin.

### Renal morphology analysis

To evaluate general morphology, Periodic Acid-Schiff (PAS) staining was performed in paraffin kidney sections (4 µm). The renal morphology was analyzed in accordance with a scoring system: a) Dilated glomerulus: 0, normal glomerular space; 1, moderately enlarged glomerular space; 2, enlarged glomerular space; 3, greatly enlarged glomerular space. b) ATN of the proximal tubule: 1, partly broken brush border, shedding and blebbing and normal nucleus; 2, > 50% broken brush border, loose cell nuclei and pyknotic nuclei; 3, more than half the tubule necrotic. c) Necrosis of the distal tubule: 1, partly loose cells; 2, > 50% loose cells and pyknotic nuclei.

### Biochemical perfusate analysis

The creatinine levels in plasma, urine, perfusion solution, and ultrafiltrate were determined by the Clinical Chemistry Laboratory at University Medical Center Groningen, in accordance with standard biochemical methods.

Creatinine Clearance (CrCl) was estimated as the glomerular filtration rate with the following formulas: a) *In vivo*: CrCl [mLmin^−1^/g] = (urine creatinine concentration [mmoL/L]. urine production flow [ml/min] / serum creatinine concentration [mmoL/L])/kidney weight [g]); b) During renal perfusion (IPK): CrCl [mLmin^−1^/g] = (ultrafiltrated creatinine concentration [µmoL/L]. ultrafiltrated production flow [ml/min] / perfusion solution creatinine concentration [µmoL/L]) / kidney weight [g]).

### Immunohistochemistry

Frozen kidney tissue sections (4 µm) were fixed in acetone (10 min), followed by endogenous peroxidase blocking (2% H_2_O_2_). Sections were incubated in TBST (supplemented with 1% BSA and rabbit antibody to C3/C3d (1:2000; Dako, USA) and mouse anti-rat-membrane attack complex (MAC/C5b-9) (1:20; Hycult Biotech, Netherlands) for 1h at Room Temperature (RT). Following washing with PBS, all sections were incubated for 1h at 37°C with goat anti-rabbit or rabbit anti-goat secondary antibodies (1:100) and the third antibody rabbit anti-goat (1:100, Dako) conjugated to peroxidase (HRP; Millipore, USA). Subsequently, the sections were washed with PBS, stained with peroxidase substrate 3-amino-9-ethylcarbazole (AEC; Sigma-Aldrich, Netherlands) for 15 min, and counterstained with hematoxylin. Negative control samples were incubated in the absence of primary antibodies.

To estimate the expression of Myeloperoxidase (MPO) and ED-1, paraffin-embedded renal tissues were sectioned (4 µm), rehydrated, incubated with citrate buffer Ph 6.0 (20 min ‒ 100°C) or EDTA pH 8.0 (15 min ‒ 95°C), respectively, to retrieve antigens. Subsequently, the sections were introduced to block nonspecific sites and endogenous peroxidase (0.3% H_2_O_2_ ‒ 30 min) and incubated at RT with rabbit antibody to rat MPO (1:20 ‒ 1h; Abcam, UK) or mouse antibody to rat CD-68 ED-1 (1:400 – 1h; Bio-Rad AbD Serotec, USA). Following washing with TBST, sections were incubated for 30 min at RT with secondary antibodies attached to horseradish peroxidase (HRP): rabbit anti-mouse (1:100, Dako) or goat anti-rabbit (1:100, Dako), and goat anti-rabbit (1:100, Dako), rinsed, stained with DAB for 15 min (Vector Laboratories, Burlingame, USA), and counterstained with hematoxylin. Negative control samples were incubated without primary antibodies.

In a parallel set of experiments, paraffin kidney sections (4 µm) were rehydrated, and incubated with citrate buffer (pH 6.0, for 20 min at 100°C), to retrieve antigens, and the non-specific sites were blocked. Subsequently, endogenous peroxidase blockage was made (0.2% H_2_O_2_ for 15 min) and after rinsing the sections received primary antibodies overnight at 4°C: anti-KIM-1 (1:50; LSBio, USA), anti-iNOS (1:500; LSBio), anti-eNOS (1:100; Boster, USA), anti-ET-1 (1:50; Santa Cruz Biotechnology, USA), anti-Caspase-3 (1:100; Abcam), Anti-Bax (1:100; Boster), anti-nNOS (1:100; Boster) and anti-BCl-2 (Abcam, 1:100). Following washing with TBS-T, all sections were incubated for 1h at 37°C with goat anti-rabbit or goat-anti mouse secondary antibodies conjugated to peroxidase (1:400; Boster). Subsequently, the sections were rinsed, stained with peroxidase substrate 3-amino-9-ethylcarbazole (AEC; Sigma-Aldrich) for 20 min, and counterstained with hematoxylin.

In all analyses performed here used an image acquisition system with a digital camera (DS-Ri1, Nikon, Japan) connected to a Nikon microscope, and evaluated using the NIS-Elements BR software (Nikon).

### Gene expression analysis

Kidney tissue samples were reserved at -80°C until total RNA was extracted using TRIzol (Invitrogen Life Technologies, USA). Real-time (RT)-PCR was performed with SYBR green PCR Master Mix (Applied Biosystems, USA) on Taqman Applied Biosystems 7900HT RT-qPCR system (Applied Biosystems), with SYBR®Green primers (Applied Biosystems) against β-actin, eNOS, iNOS, Caspase-3, BCL-2, and KIM-1 (Appendix). The amplification cycle was initiated with one cycle at 50°C (2 min) and 95°C (10 min), followed by 40 cycles at 60°C (15s) and 60°C (1 min). Cycle Threshold (CT) values were corrected with the housekeeping gene (β-actin). Relative gene expression was established with reference to sham animals' reference media value for each gene (*n* = 6‒8) of the respective sex.

### Statistical analysis

Data were expressed as the mean ± Standard Error of the Mean (SEM) or as the median and 95% percentile interval (gene expression data). Data were analyzed with GraphPad Prism Software V9. In relation to renal perfusion, male and female groups, before and after renal perfusion, were compared with two-way, followed by two-stage step-up mode of Benjamini, Krieger and Yekutieli test or the Mann-Whitney test for multiple comparisons, in abnormally distributed data (gene expression data). Statistical significance was set at *p* < 0.05.

## Results

### Renal morphology

**Fig. 2:**
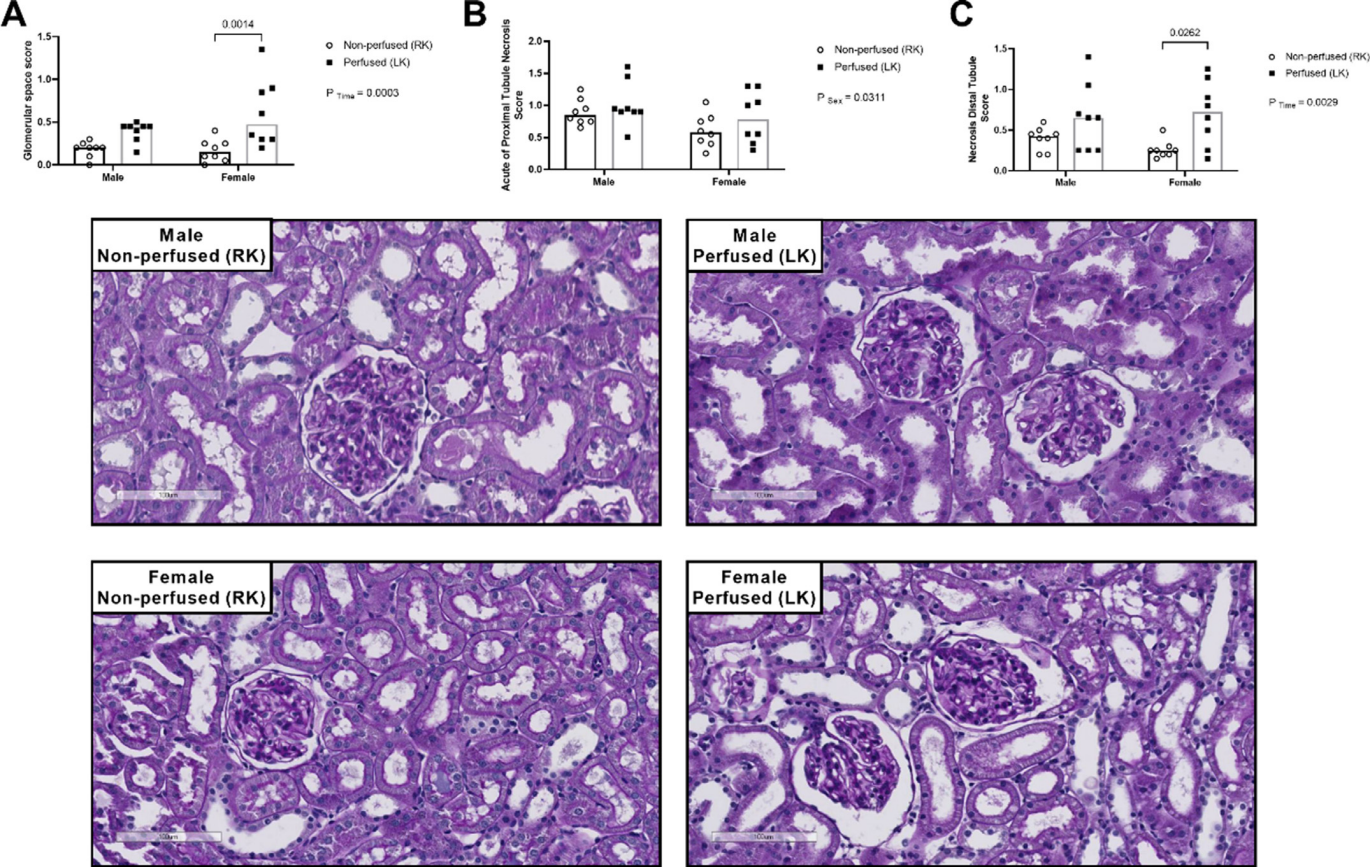
Sex differences in the renal morphology. (A) glomerular space score; (B) acute of proximal tubule necrosis score; (C) necrosis distal tubule score. Rats were submitted to BD and kidneys placed in the renal perfusion (IPK ‒ 90min). RK, Right Kidney, non-perfused; LK, Left Kidney, perfused. Data expressed as mean ± SEM (*n* = 6‒8 per group, 1 tissue section per animal, 5 fields).

Regarding glomerular morphology, in comparison to respective non-perfused kidney, male and female perfused kidneys presented a higher glomerular space score (Fig. 2A).

In relation to renal tissue necrosis, BD-male kidneys showed higher values of acute proximal tubule necrosis before and after renal perfusion (Fig. 2B). Additionally, both sexes had higher scores of necrosis distal tubule, in comparison to respective non-perfused kidneys (Fig. 2C).

### Protein and gene expression of cell death mediators

**Fig. 3:**
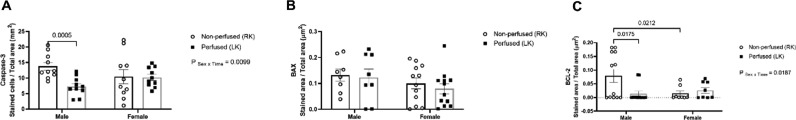
Sex differences in the protein expression of Caspase-3 (A), BAX (B) and BCL-2 (C) in renal tissue. Rats were submitted to BD and kidneys placed in the renal perfusion (IPK ‒ 90min). RK, Right Kidney, non-perfused; LK, Left Kidney, perfused. Data expressed as mean ± SEM (*n* = 6‒8 per group, 1 tissue section per animal, 5 fields).

BD-male kidneys presented lower values of Caspase-3 protein expression after renal perfusion (Fig. 3A).

No considerable differences were found between sexes in the BAX protein expression in the renal tissue (Fig. 3B).

In regard to the anti-apoptotic protein BCL-2, non-perfused male kidneys presented higher BCL-2 than females. After perfusion, male kidneys presented a reduction in BCL-2 protein expression and are similar to females (Fig. 3C).

**Fig. 4:**
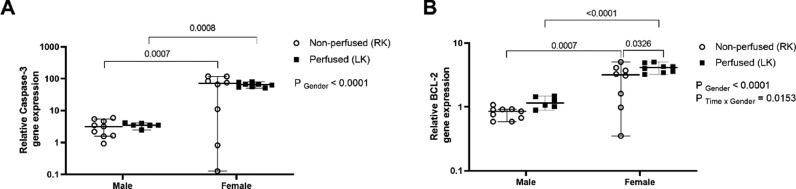
Sex differences in the gene expression of Caspase-3 (A), and BCL-2 (B) in renal tissue. Rats were submitted to BD and kidneys placed in the renal perfusion (IPK ‒ 90 min). RK, Right Kidney, non-perfused; LK, Left Kidney, perfused. Data expressed as median and 95% percentile interval (*n* = 6‒8 per group).

Regarding gene expression of apoptosis mediators, kidneys from female rats showed upregulation of Caspase-3 after BD in relation to kidneys from male rats, which was maintained after renal perfusion (Fig. 4A). In regard to BCL-2 gene expression, kidneys from female rats showed upregulation of BCL-2 before and after renal perfusion, in comparison to the kidneys from male rats (Fig. 4B).

### Renal function

**Fig. 5:**
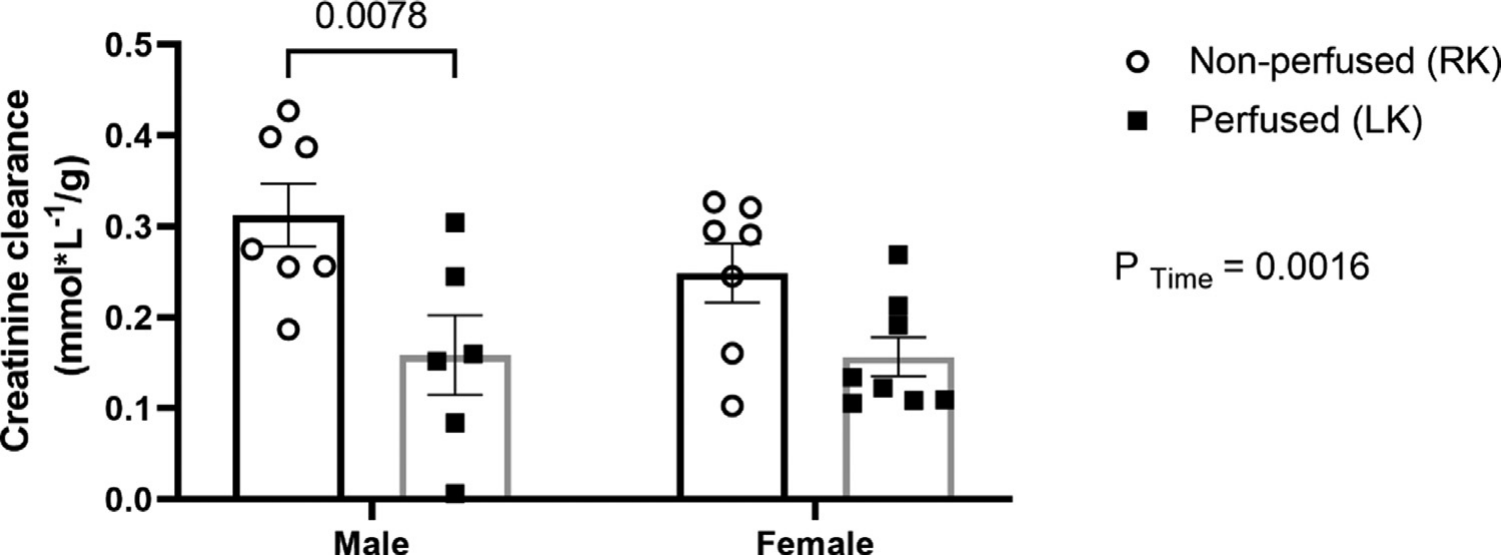
Sex differences in creatinine clearance during renal perfusion (IPK). Rats were submitted to BD and kidneys placed in the renal perfusion (IPK ‒ 90 min). RK, Right Kidney, non-perfused; LK, Left Kidney, perfused. Data expressed as mean ± SEM (*n* = 6‒8 per group).

There was a decline in creatinine clearance in both sexes (Fig. 5).

### Kidney injury marker – 1 (KIM-1) protein and gene expression

**Fig. 6:**
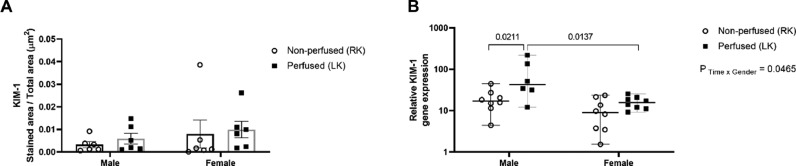
Sex differences in protein (A) and relative gene expression (B) of KIM-1 in renal tissue. Rats were submitted to BD and kidneys placed in the renal perfusion (IPK ‒ 90 min). RK, Right Kidney, non-perfused; LK, Left Kidney, perfused. Data expressed as mean ± SEM (A) and median and 95% percentile interval (B) (*n* = 6‒8 per group, 1 tissue section per animal, 5 fields).

Despite the higher values found in BD-female kidneys, no significant difference was found before or after renal perfusion (Fig. 6A). Additionally, only kidneys from male rats showed an upregulation of KIM-1gene after renal perfusion (Fig. 6B).

### Renal perfusion flow

**Fig. 7:**
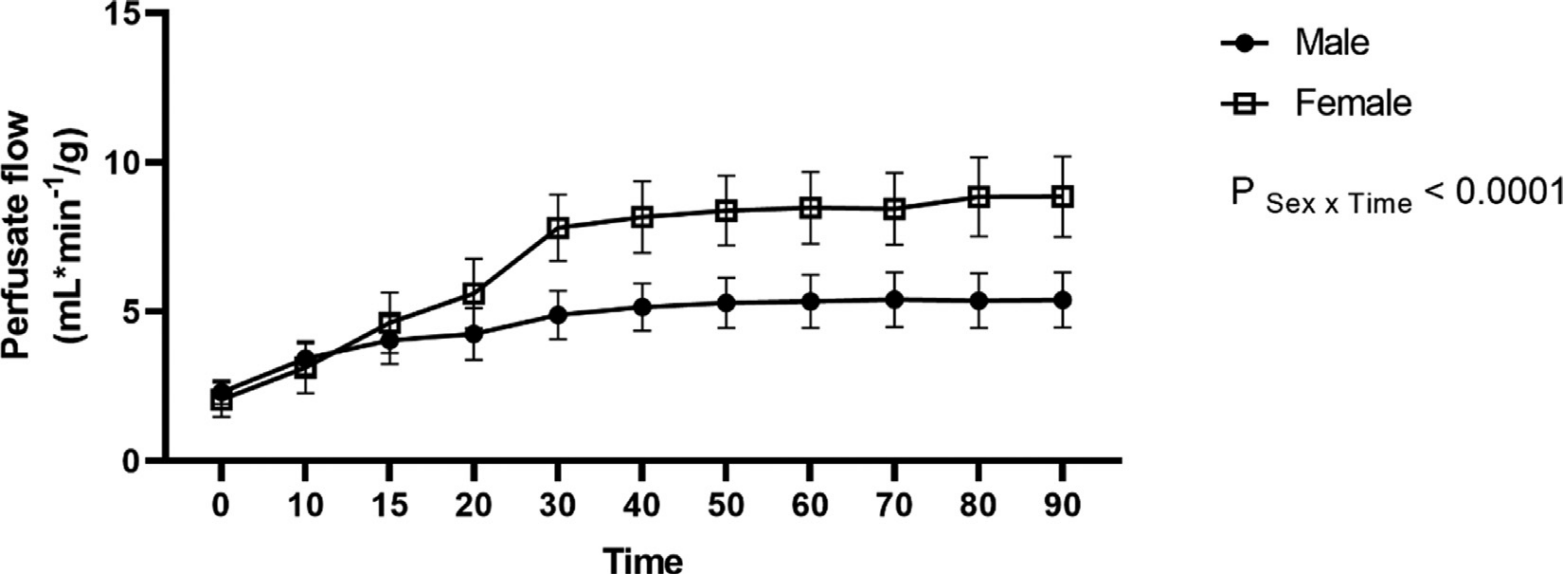
Sex differences in perfusate renal flow per minute per gram. Rats were submitted to BD and kidneys placed in the renal perfusion (IPK ‒ 90 min). RK, Right Kidney, non-perfused; LK, Left Kidney, perfused. Data expressed as mean ± SEM (*n* = 6‒8 per group).

Renal flow was significantly lower in BD-male kidneys, in comparison to BD-female kidneys (Fig. 7).

### Nitric oxide synthase (NOS) isoforms and endothelin-1 (ET-1) expression

**Fig. 8:**
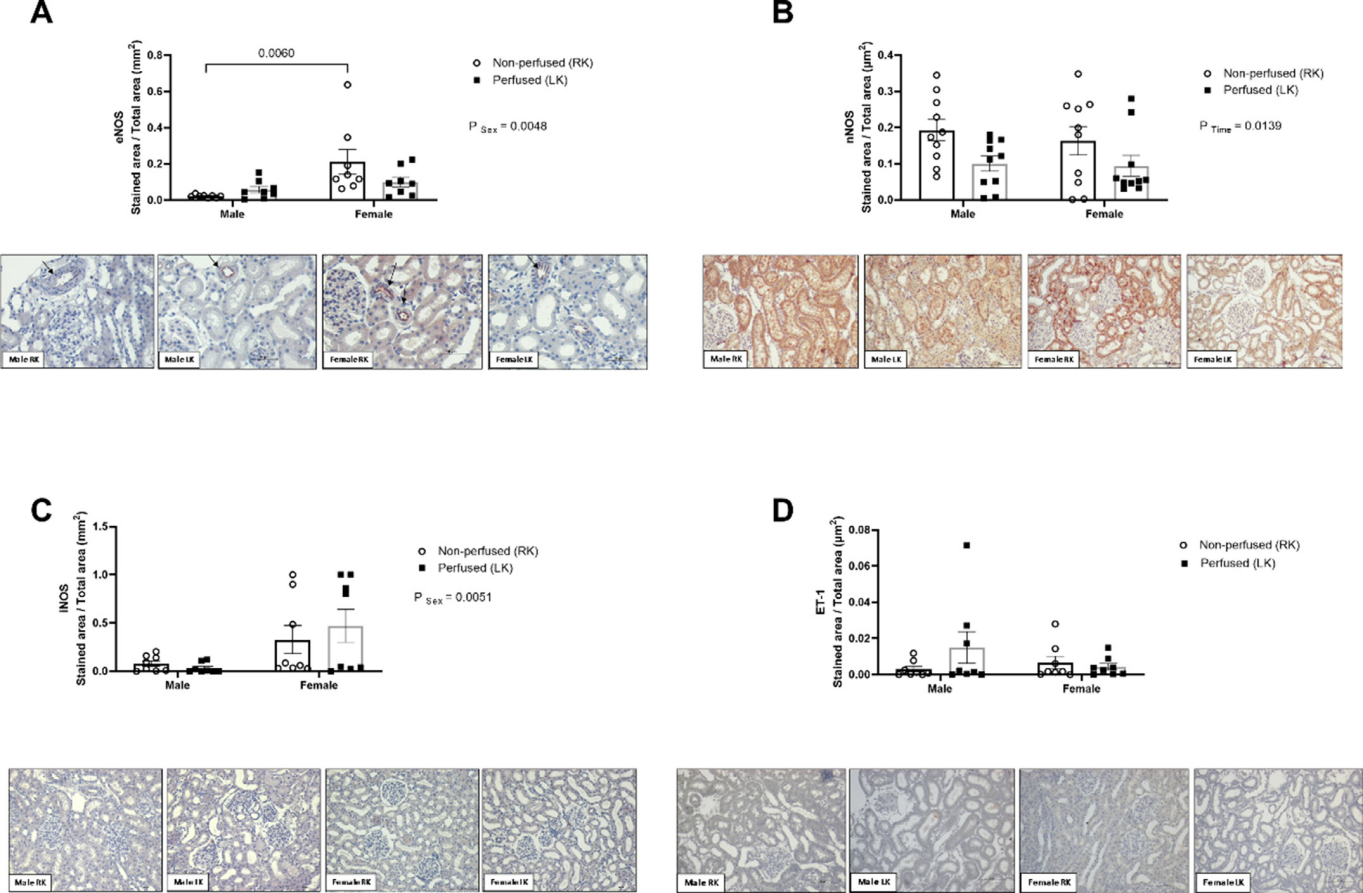
Immunostaining for protein expression of eNOS (A), nNOS (B), iNOS (C) and ET-1 (D) in renal tissue. Rats were submitted to BD and kidneys placed in the renal perfusion (IPK ‒ 90 min). RK, Right Kidney, non-perfused; LK, Left Kidney, perfused. Arrows point the immunohistochemical reaction of eNOS. Original magnification images: 40 × for eNOS, 20 × for nNOS, iNOS and ET-1. Data expressed as mean ± SEM (*n* = 6‒8 per group, 1 tissue section per animal, 5 fields).

eNOS expression was significantly higher in BD-female kidneys before renal perfusion (Fig. 8A).

With respect to nNOS protein expression in the renal tissue, BD male and BD female kidneys presented reduction after renal perfusion (Fig. 8B). iNOS protein expression was higher in BD-female kidneys before and after renal perfusion (Fig. 8C). In the analysis of ET-1 protein expression, no important differences were evidenced between the sexes (Fig. 8D).

**Fig. 9:**
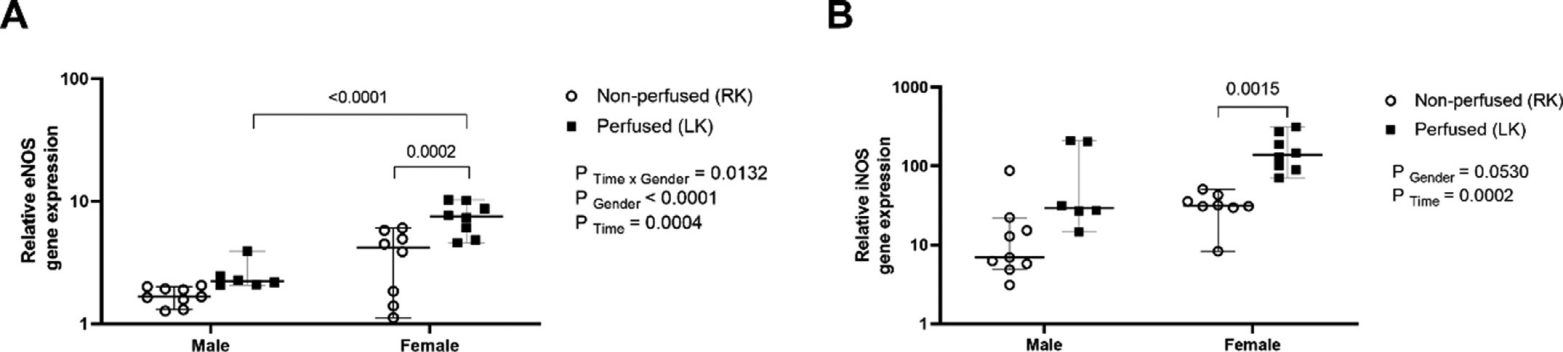
Sex differences in the relative gene expression of eNOS (A) and iNOS (B) in renal tissue. Rats were submitted to BD and kidneys placed in the renal perfusion (IPK ‒ 90 min). RK, Right Kidney, non-perfused; LK, Left Kidney, perfused. Data expressed as median and 95% percentile interval (*n* = 6‒8 per group).

Regarding gene expression, kidneys from female rats showed upregulation of eNOS before and after renal perfusion, in relation to the kidneys from male rats (Fig. 9A). In addition, iNOS gene expression was upregulated in BD-female kidneys compared to that in non-perfused kidneys from the same group (Fig. 9B).

### Complement system mediators and inflammatory cells

Table 1 summarizes the inflammatory cellular profile and complement system mediators in the renal tissue.

**Table 1:** Complement system mediators and inflammatory cells.

	Renal perfusion						
	Male		Female		P Anova		
	Non-perfused (RK)	Perfused (LK)	Non-perfused (RK)	Perfused (LK)	P _Sex × Time_	P _Sex_	P _Time_
**MPO Stained cells/Total area (mm^2^)**	0.042 ± 0.009	0.038 ± 0.020	0.031 ± 0.009	0.034 ± 0.012	0.4754	0.1764	0.9458
**ED-1 Stained cells/Total area (mm^2^)**	0.094 ± 0.024	0.141 ± 0.057^a^	0.078 ± 0.033	0.090 ± 0.031	0.1435	0.0606	0.0192
**C3/C3d Stained area / Total area**	0.046 ± 0.008	0.065 ± 0.013^a^	0.059 ± 0.010	0.058 ± 0.010	0.0178	0.5368	0.0384
**C5b-9 (membrane attack complex) Stained area / Total area**	0.013 ± 0.009	0.082 ± 0.047^a^	0.016 ± 0.008	0.061 ± 0.035^a^	0.3358	0.3691	0.0005

Data expressed as mean ± SEM (*n* = 7). Statistical testing consisted of ANOVA followed by Dunnett test for multiple comparison. RK, Right Kidney, LK, Left Kidney.

a *p* < 0.05 in relation to non-perfused kidney.

Mainly expressed by neutrophils, MPO expression was higher in BD-male kidneys before renal perfusion. After renal perfusion, no significant differences were observed between the sexes. There were no significant differences trigged by BD in the ED-1 expression between the sexes. After renal perfusion, BD-male and BD-female kidneys presented higher values for ED-1 expression.

Regarding complement system activation, BD caused higher C3/C3d deposition in female kidneys. But after renal perfusion, only BD-male kidneys showed high C3/C3d deposition. No sex differences in the development of C5b-9 after BD were observed. After renal perfusion, both sexes presented higher values in the development of C5b-9.

## Discussion

Previous studies have shown that female rats subjected to Brain Death (BD) exhibit greater cardiac, pulmonary, and intestinal inflammatory response than males, which correlates to the sudden reduction of Female Sex Hormones (FSH) after BD induction [3,8,11]. Here, the authors extended the investigation of sex influence on the detrimental process triggered by BD in a normothermic Isolated Perfused Kidney (IPK) setup. The technique allows the evaluation of renal function without systemic influences, such as blood pressure, inflammatory mediators, or hormones.

The renal microvasculature exerts a key aspect in the pathophysiology of Acute Kidney Injury (AKI) [12]. The results presented here evidence significant sex differences in the preservation of the renal perfusion, evidenced in the perfusate flow in the IPK. Kidneys from female donors submitted to BD did not show a reduction of perfusate flow as found in BD-male kidneys in the IPK. Previous experimental studies showed that, after BD, there is an immediate reduction in the perfusion of microvessels (diameter ≤ 30 μm) [13]. The preservation of renal perfusion by female rats, as evidenced here, is in line with the recognized cardiovascular influence of estradiol [14] and its role in blood vessel dilation and amelioration in sepsis and trauma experimental models [15,16]. As mentioned previously, the female group presented in the present study used rats under elevated estradiol secretion stage at the period of BD establishment. The maintenance of organ perfusion in BD-female rats at the stage of high estradiol concentration of the estrous cycle has also been shown in other models [17]. In addition, Ferreira et al. [3] showed that BD led to hypoperfusion of mesenteric microvessels in male rats, but not in female rats. Such a result was associated with greater endothelial Nitric Oxide Synthase (eNOS) protein expression in female rats after BD. In this context, the authors also saw higher protein expression of eNOS in the kidneys of BD-female rat donors, in relation to that in the kidney of BD-male rats, which could be a consequence of estradiol maximal concentration in female rats previous to BD induction. The sustained eNOS protein expression, despite the common decline of estradiol provoked by BD seen in previous studies [18,19] might maintain the NO synthesis, supporting the regulation of vascular tonus and consequently preserving renal perfusion. Alternatively, the reduction of eNOS and the Neuronal Nitric Synthase Oxide (nNOS) protein expression after renal perfusion in the female kidneys could suggest an eventual reduction of perfusate flow after longer periods of renal perfusion (IPK).

In the course of hypoperfusion/ischemic time, tissues are deprived of oxygen/nutrients required to preserve cellular homeostasis. Sublethal injury leads to the breakdown of cell polarity, as disruption of cytoskeletal integrity, relocalization of membrane proteins/adhesion molecules, and cells could recuperate if the insult is not terminal, whereas more severe injury results in irremediable renal tubular cell death by apoptosis and/or necrosis, culminating in renal dysfunction seen in AKI [20]. Tubular Epithelial Cells (TECs) death is a classic indicator of AKI [21]. Upregulation of caspase-3, the key effector of apoptosis, has been evidenced in renal tubular and microvascular endothelial cells in the early phases of AKI [22]. In addition, in acute injuries, such as ischemia/reperfusion models, the proapoptotic molecule Bax in the TEC turns activated, leading to the depletion of the antiapoptotic protein BCL-2, and thus altering the pro-/antiapoptotic mechanism (i.e., Bax/Bcl-2 ratio) equity towards the initiation of apoptosis [23]. Necrotic cell death in acute injuries is credited to generally occur secondary to mitochondrial permeability transition pore development, which consists of the multimetric protein complex F1/F0 ATP-synthase, on the inner mitochondrial membrane and Bax/Bak on the external membrane [24]. In the present study, BD induction triggered apoptosis in both sexes, evidenced by higher values of pro-apoptotic caspase-3 and BAX. However, the authors suggest that ex-vivo perfusion of kidneys in the IPK setup maintains the state of the organ, the pro-inflammatory environment, and the apoptosis pathway triggered by BD resulting in an exuberant process of renal necrosis in the kidneys from the male group. Necrosis is an uncontrolled condition of cell death that is triggered by external injury, such as inflammation or hypoxia [25]. In parallel, despite no significant changes observed in protein expression of the Kidney Injury Marker-1 (KIM-1), BD-male kidneys presented upregulation of KIM-1 gene expression. Clinical and experimental evidence indicates that KIM‐1 mRNA levels are increased more than any known gene in both humans and rodents following kidney injury [26,27].

BD triggers systemic and local inflammatory cascades, including complement system and endothelial cell activation, inflammatory mediators release (chemokines and cytokines), and leukocyte infiltration into the organs 28, 29, 30. In BD-organ donors, the complement system is activated systemically and locally and is a critical cascade of inflammation and graft injury [31,32]. BD repercussions contribute to the formation and influx of Damage-Associated Molecular Patterns (DAMPs) into the systemic and local circulation [31]. DAMPs induce activation of the complement system, leading to an inflammatory response, culminating in the formation of anaphylatoxins such as C3a and C5a. Afterward, final cascade activation results in the development of the membrane attack complex (MAC/C5b-9). Similar data was seen in the present study, as male and female rats submitted to BD evidenced deposition and formation of C3 and C5b-9 in the kidneys after 90 min of renal perfusion. The release of DAMPs and complement mediators is identified by cellular receptors on monocytes, neutrophils, and other immune cells, resulting in leukocyte activation. Concurrently, these repercussions cause endothelial and tissue damage, leading to lower organ viability [33]. Here, the proinflammatory environment triggered by BD in males and females resulted in the augmentation of macrophage activation, evidenced by the increase of ED-1 staining cells after renal perfusion. It is important to highlight that macrophages are crucial inflammatory cells participating in the whole phase of AKI [34].

The progression of renal injury leads to the loss of renal function. AKI is a common clinical issue evidenced by reduced Glomerular Filtration Rate (GFR) [35]. Previous experimental studies evidenced that the renal function is negative in the course of 4h of BD followed by suboptimal results succeeding reperfusion in an IPK setup [36]. A similar effect was evidenced here, as the gradual renal injury triggered by BD led to a significant reduction in creatinine clearance in both the BD-male and BD-female groups.

Although the main point of the present study is the emphasis on the sex differences in BD-related renal injury, the lack of kidney transplantation in the experimental model represents a limitation. Furthermore, the authors made use of an investigation time of 4h, which is frequently the standard duration in this type of experimental protocol.

## Conclusion

Considering that male and female rats were subjected to IPK and that BD-induced effects evolve over time, the differences in renal injury and inflammation between sexes may be accentuated following longer periods of BD and IPK. In this context, the findings presented here suggest as the main sex difference is the microcirculatory response to BD and IPK, especially in the preservation of renal perfusion and vascular eNOS. In relation to inflammation and function, both sexes suffer from deterioration due to BD and IPK time. Since the therapeutic with 17β-estradiol has been effective in protecting several organs from deleterious effects triggered by BD [18,19,37, 38, 39], further studies with ex *vivo* perfusion may incorporate 17β-estradiol in the perfusate solution as a prospective therapy to improve the renal graft of both sexes for transplantation.

## Authors’ contributions

R.A.Jr.: Conceptualization, Data curation, Formal analysis, Writing - original draft, review and editing. F.Y.R.S., M.V.S., L.F.A., P.J.O. and C.J.C. Data curation, Methodology, Validation and Visualization H.G.D.L., L.F.P.M.: and. A.C.B.F.: Conceptualization, Funding acquisition, Surpervision, Writing - original draft and review and editing.

## Availability of data and materials

The inquiries regarding the original data presented in the study can be directed to the corresponding authors.

## Declaration of Competing Interest

The authors declare no conflicts of interest.

## Appendix Supplementary materials

Image, application 1
