# Supplementary material for: Comparison of acute kidney injury following brain death between male and female rats
Source: Clinics (Sao Paulo). 2023 May 31;78:100222. doi: 10.1016/j.clinsp.2023.100222 (PMC10244907; doi:10.1016/j.clinsp.2023.100222)
Supplement: Supplementary file 1 [file mmc1.docx]

CLINICS-D-23-00038_Supplementary Material

**Appendix** Gene specific qPCR primers and their respective PCR fragment lengths.

| **Primer** | **Foward primer sequencies** | **Reverse primer sequencies** |
| --- | --- | --- |
| β-actin | 5’-GGAAATCGTGCGTGACATTAAA-3’ | 5’-GCGGCAGTGGCCATCTC-3’ |
| eNOS | 5’-GAGGAGCCCAAAGGCACAAG-3’ | 5’-CCAAACCCCTCACTGTCATTTTATT-3’ |
| iNOS | 5’-GAGGAGCCCAAAGGCACAAG-3’ | 5’-CCAAACCCCTCACTGTCATTTTATT-3’ |
| Caspase-3 | 5’-GCATGCCAGAAGATACCAGTGG-3’ | 5’-AGTTTCAGCATGGCGCAAA-3’ |
| BCL-2 | 5’-CTGGGATGCCTTTGTGGAA-3’ | 5’-TCAGAGACAGCCAGGAGAAATCA-3’ |
| KIM-1 | 5’-AGAGAGAGCAGGACACAGGCTTT-3’ | 5’-ACCCGTGGTAGTCCCAAACA-3’ |

eNOS, Endothelial Nitric Oxide Synthase; iNOS, Inducible Nitric Oxide Synthase; BCL-2, B-Cell Leukemia/Lymphoma-2; KIM-1, Kidney Injury Molecule-1.
